# Work Motivation of Medical Personnel Under Conditions of Socioeconomic Asymmetry

**DOI:** 10.1192/j.eurpsy.2026.11652

**Published:** 2026-07-31

**Authors:** S. D. Tomilovskiy, A. M. Konovalova

**Affiliations:** 1 Faculty of Psychology, Lomonosov Moscow State University; 2 Department of Pedagogy and Medical Psychology, Sechenov First Moscow State Medical University, Moscow, Russian Federation

## Abstract

**Introduction:**

Work motivation of medical personnel is a critical resource for the resilience of mental health care systems. Socioeconomic inequality between regions may significantly affect the structure of this motivation: differences in salary levels, technical resources, and organizational culture can shape fundamentally different behavioral orientations among staff. However, regional disparities in the motivational profiles of healthcare professionals remain insufficiently studied.

**Objectives:**

To compare the structure of occupational motivation among medical workers employed in public healthcare institutions operating under contrasting socioeconomic conditions.

**Methods:**

The study involved 80 participants: 40 employees from public medical institutions in Moscow and 40 from the Stavropol region. Two validated instruments were used: the “Work Motivation Inventory” by K. Zamfir (adapted by A. A. Rean) to assess types of professional motivation, and V. E. Milman’s “Motivational Personality Structure Inventory.” Statistical analysis was conducted using the Mann–Whitney U test.

**Results:**

Both groups demonstrated a functional motivational profile (internal > external positive > external negative motivation). However, the regional group showed significantly higher levels of external negative motivation (p = 0.044) and greater emphasis on the motive of social status (p < 0.01). Conversely, the capital city group demonstrated significantly stronger comfort-related and creative activity motives (p < 0.05). No significant differences were found in levels of internal motivation.

**Conclusions:**

The socioeconomic context exerts a significant influence on the structure of occupational motivation in medical personnel. In resource-constrained regions with limited professional development opportunities, workers tend to rely more heavily on external behavioral regulation and seek greater social recognition. These differences should be taken into account when designing motivational strategies and human resource policies in mental health care systems, particularly at the regional level.

**Disclosure of Interest:**

None Declared

